# Case Report: Rapid improvement with upadacitinib in a case of paradoxical skin lesions associated with SAPHO syndrome

**DOI:** 10.3389/fimmu.2026.1818853

**Published:** 2026-06-04

**Authors:** Jinwan Du, Yirou Gao, Shaohui Geng, Chen Li, Shike Shang

**Affiliations:** 1 Liang Jiang Hospital of Chongqing Medical University, Chongqing, China; 2 School of Chinese Medicine, Beijing University of Chinese Medicine, Beijing, China; 3 School of Life Science, Beijing University of Chinese Medicine, Beijing, China; 4 Shanghai Guanghua Hospital of Integrated Traditional Chinese and Western Medicine, Shanghai, China

**Keywords:** Janus kinase inhibitors, rare case, SAPHO syndrome, spondyloarthritis, tumor necrosis factor-alpha inhibitors, upadacitinib

## Abstract

SAPHO syndrome is a rare autoinflammatory disorder with osteoarticular and cutaneous involvement. Paradoxical skin lesions have been reported during treatment with tumor necrosis factor-α (TNF-α) inhibitors. We describe a 42-year-old man with a 6-year history of SAPHO syndrome presenting with chronic sternoclavicular and lumbosacral pain and sterile pustules on the lower extremities. Conventional therapy with etoricoxib and methotrexate improved musculoskeletal symptoms but failed to resolve skin lesions, while adalimumab treatment led to progressive worsening of cutaneous manifestations. Following a switch to upadacitinib (15 mg once daily), rapid improvement of skin lesions and sustained control of musculoskeletal symptoms were achieved without adverse events. This case suggests that Janus kinase inhibition may represent a therapeutic option for TNF-α inhibitor–induced paradoxical skin lesions in SAPHO syndrome.

## Introduction

SAPHO syndrome is an autoinflammatory disorder characterized by synovitis, acne, pustulosis, hyperostosis, and osteitis, primarily manifesting as chronic aseptic osteoarticular lesions and dermatological involvement. In recent years, TNF-α inhibitors have proven effective for treating SAPHO syndrome. However, studies indicate they are associated with cutaneous complications, with paradoxical skin lesions being the most common dermatologic side effect (1). Current treatment approaches for biologic-induced paradoxical skin lesions vary. Here, we report a case of rapid improvement in TNF-α-induced paradoxical skin lesions of SAPHO syndrome following treatment with upadacitinib.

## Case presentation

**Figure 1:**
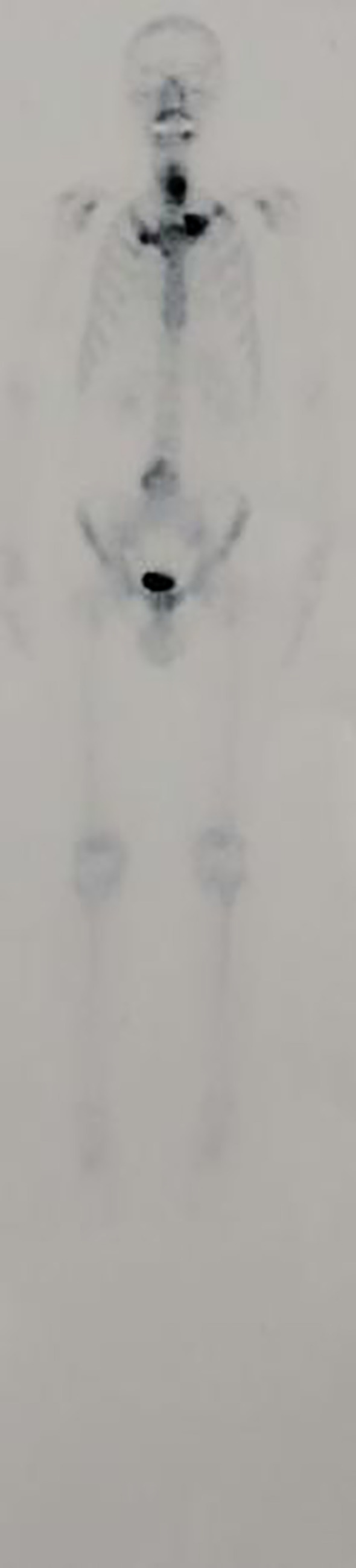
Patient bone scan image.

A 42-year-old man with a 6-year history of SAPHO syndrome presented with persistent bilateral sternoclavicular and lumbosacral pain (numerical rating scale score: 6/10), accompanied by sterile pustules on both lower legs and feet. Whole-body bone scintigraphy revealed increased uptake in the cervical spine, bilateral sternoclavicular joints, and the left clavicle (**Figure 1**). During the previous years, the patient had intermittently received symptomatic treatment with NSAIDs (mainly ibuprofen as needed) and topical corticosteroids for cutaneous symptoms, but had never received biologic agents or conventional synthetic DMARDs consistently.

At the time of diagnosis, laboratory tests demonstrated mildly elevated inflammatory markers, mainly reflected by an elevated erythrocyte sedimentation rate (26 mm/h, normal 0–15 mm/h), while C-reactive protein levels remained within the normal range (4.17 mg/L, normal 0–5 mg/L). Autoantibody screening, including antinuclear antibody (ANA), anti-double-stranded DNA (anti-dsDNA), anti-extractable nuclear antigen (anti-ENA), rheumatoid factor (RF), anti-cyclic citrullinated peptide (anti-CCP), and anti-neutrophil cytoplasmic antibody (ANCA), was negative. Liver and kidney function were normal, and no evidence of infection or systemic autoimmune disease was identified. The patient had no significant comorbidities (e.g., diabetes, hypertension, or chronic infections) and was not taking any other concurrent medications.

**Figure 2:**
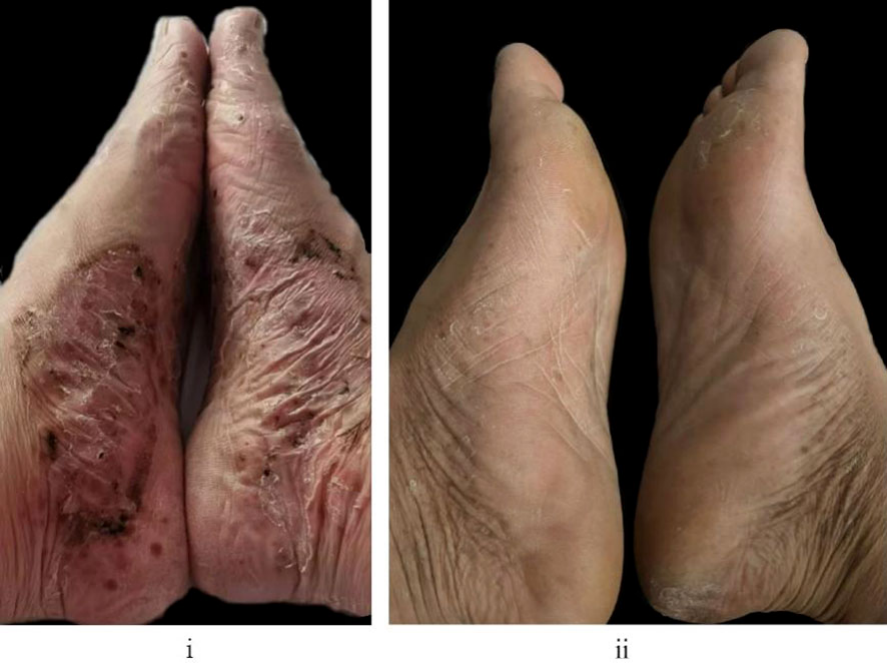
Cutaneous manifestations before and after treatment switch. i) Bilateral foot lesions after 2 months of adalimumab treatment; ii) Bilateral foot lesions after upadacitinib treatment.

**Figure 3:**
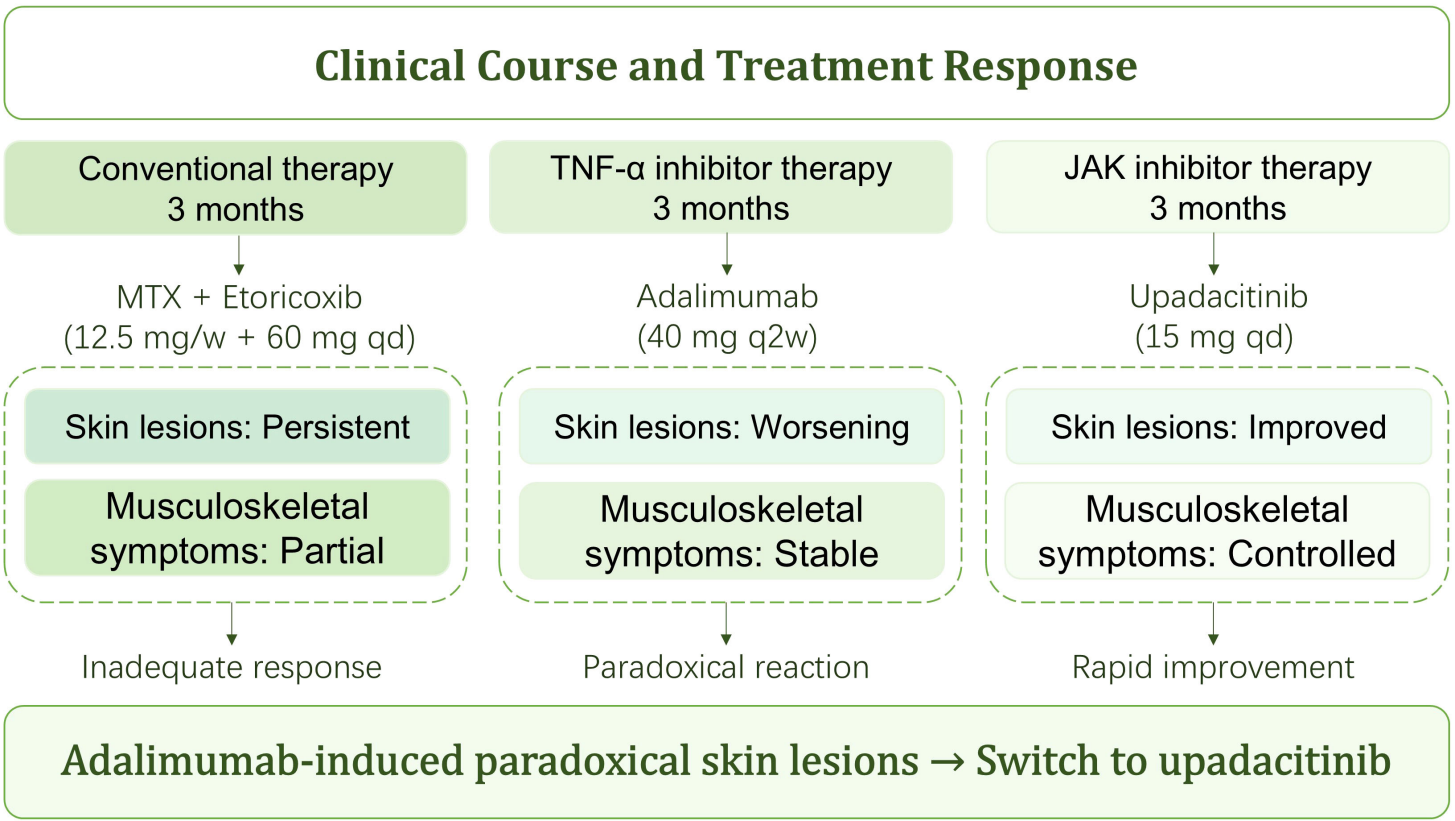
Clinical course and treatment response of the patient.

Based on the clinical manifestations and imaging findings, the patient was initially treated with etoricoxib (60 mg once daily) and methotrexate (12.5 mg weekly). The methotrexate dose was suboptimal relative to standard protocols (usually 15–25 mg weekly) due to the patient’s concerns about tolerability. After 3 months, joint pain improved partially, whereas skin lesions persisted. Subsequently, adalimumab (40 mg every 2 weeks) was initiated. However, after 3 months, the skin lesions progressively worsened, with the development of eczematous changes. The lesions were symmetrically distributed on the medial aspects of both feet, presenting as polymorphic eruptions on an erythematous base with indistinct borders, including papules, papulovesicles, and vesicles, some coalescing into plaques, accompanied by exudation, crusting, and mild scaling (**Figure 2**). Subsequently, the patient stopped adalimumab and switched to upadacitinib (15 mg once daily). After 3 months, the cutaneous lesions improved markedly, and the patient reported significant symptom relief (**Figure 2**). The treatment was well tolerated, with no adverse events observed during therapy or the 3-month follow-up. Laboratory parameters, including liver and kidney function, remained within normal ranges. The clinical course is summarized in **Figure 3**.

## Discussion

The pathogenesis of SAPHO syndrome remains unclear. Studies suggest it involves the release of multiple inflammatory cytokines, particularly the overexpression of proinflammatory cytokines TNF-α and IL-17 (2).In recent years, TNF-α inhibitors have been employed to treat SAPHO patients unresponsive to conventional therapies or with refractory disease. A cohort study of 164 SAPHO patients documented adverse effects of TNF-α inhibitor therapy, with 7 out of 41 treated patients (17.1%) ultimately developing paradoxical skin lesions (3). The currently hypothesized mechanism suggests that IFN-γ plays a potent activating role in TNF-α inhibitor-induced paradoxical skin lesions. Elevated IFN-γ levels are primarily produced by Th1 lymphocytes, representing a response to the massive IFN-γ production by plasma cell-like dendritic cells activated by TNF blockade (4).Additionally, IL-17 released from Th17 cells has been identified as a potential trigger in the pathogenesis of skin lesions (5).

Multiple studies have now demonstrated the efficacy of JAK inhibitors in SAPHO syndrome (6, 7). The JAK pathway is critical for immune responses, with numerous cytokines mediated through JAK, such as IFN-α, IL-6,IL-7, among others. IL-6 regulates immune cells like T cells to produce IL-17 via the JAK pathway. Both IFN-α and IL-17 play pivotal roles in TNF-α-induced atypical skin lesions of SAPHO syndrome (8). The JAK/STAT pathway, formed by JAKs and downstream activated STATs, constitutes a major signaling pathway in inflammatory diseases. JAK inhibitors (including JAK1, JAK2, JAK3, etc.) effectively block upstream components and pathways of various cytokines. Compared to biologics targeting single cytokines, JAK inhibitors exhibit more pronounced anti-inflammatory activity (9) and can further improve skin lesions.

Upadacitinib is a novel, selective, orally administered, reversible JAK1 inhibitor (10) with minimal effects on other JAK subtypes. It selectively inhibits individual JAK proteins without affecting other cytokines, thereby achieving therapeutic goals. Previous studies have primarily reported the use of JAK inhibitors such as tofacitinib and baricitinib in refractory SAPHO syndrome, showing favorable outcomes. For instance, Yang et al. described a patient refractory to NSAIDs, DMARDs, and TNF-α inhibitors who achieved significant improvement after 4 weeks of tofacitinib therapy (11). Furthermore, a recent systematic review involving 72 SAPHO patients showed that approximately 98.6% of patients demonstrated good or complete efficacy after receiving JAK inhibitor therapy, further supporting their potential efficacy in refractory SAPHO (12).

Compared with previous reports, this case highlights a distinct clinical scenario in which JAK inhibitor therapy was used to manage TNF-α inhibitor–induced paradoxical skin lesions rather than refractory osteoarticular symptoms alone. The rapid improvement in both cutaneous and musculoskeletal manifestations suggests that upadacitinib may be particularly effective in correcting cytokine imbalances associated with TNF inhibitor–induced immune dysregulation.

Despite their efficacy, JAK inhibitors are associated with potential safety concerns. Inhibition of the JAK/STAT pathway may impair host immune defense, increasing the risk of infections, including opportunistic infections and viral reactivation (e.g., herpes zoster). Additional adverse effects include hematologic abnormalities and dyslipidemia, and long-term use may be associated with cardiovascular risk (13). Therefore, careful risk–benefit assessment and regular monitoring are essential in clinical practice. In this case, the patient was fully informed of potential risks prior to treatment initiation and provided informed consent.

This study has several limitations. First, as a single case report, its findings are not generalizable. Second, the follow-up duration was limited to 3 months, precluding assessment of the long-term efficacy and safety of upadacitinib therapy. Longer-term follow-up data were not available in the present case. Third, histopathological examination of the skin lesions was not performed, limiting precise classification of the cutaneous manifestations.

## Conclusion

In summary, we report a case of TNF-α inhibitor–induced paradoxical skin lesions in SAPHO syndrome successfully treated with upadacitinib. Both cutaneous and osteoarticular symptoms improved rapidly without adverse events. This finding suggests that JAK inhibitors may represent a promising therapeutic option for managing biologic-induced immune dysregulation in SAPHO syndrome.

## Data Availability

The original contributions presented in the study are included in the article/supplementary material. Further inquiries can be directed to the corresponding authors.
